# Effects of Au and Ge Additions on the Microstructures and Properties of Ag-1.5Cu-0.1Y Alloys

**DOI:** 10.3390/ma12010123

**Published:** 2019-01-02

**Authors:** Desheng Zhang, Qin Zhang, Sida Li, Hongying Yang

**Affiliations:** School of Metallurgy, Northeastern University, Shenyang 110819, China; zhangdesheng1988@126.com (D.Z.); zhangq@smm.neu.edu.cn (Q.Z.); lisida1024@163.com (S.L.)

**Keywords:** Ag-1.5Cu-0.1Y alloy, Au, Ge, microstructure, corrosion resistance, microhardness, conductivity

## Abstract

The application of silver is seriously affected by its tendency to oxidize and corrode. Therefore, the addition of proper alloying elements to silver-based alloys to achieve better properties has become a hot topic at present. In this current study, the effects of the addition of the two elements Au and Ge on the microstructures and properties of Ag-1.5Cu-0.1Y alloys were investigated. The results showed that the microstructures were refined and the second dendrite was shortened in the Ag-1.5Cu-0.1Y alloys with the addition of Au and Ge. Adding Au enhanced the corrosion resistance of the Ag-1.5Cu-0.1Y alloys. Furthermore, the corrosion resistance of the Ag-1.5Cu-0.1Y alloys with the addition of both Ge and Au was better than that of the alloy samples with Au added alone. The best corrosion resistance of the Ag-1.5Cu-0.1Y alloys was achieved by adding 1.0 wt.% Au and 1.0 wt.% Ge. The microhardness was enhanced by the addition of Au and Ge, and was strongly correlated with the secondary dendrite arm spacing (λ_2_) of the Ag-1.5Cu-0.1Y alloys. In addition, the Au addition could improve the conductivity of the Ag-1.5Cu-0.1Y alloy; however, Ge had little effect on the conductivity of the alloy samples. This work provides an experimental basis for the design of better performing silver-based alloys.

## 1. Introduction

Silver (Ag), as a precious metal, has a low price and bright white color. Therefore, it gained high popularity in ancient China and was widely used in the production of money, tableware, ornaments, works of art, etc. [1,2]. Silver is the metal with the highest reflectivity and thus is widely used in the preparation of targets, optical recording medium reflective films [3,4], semi-transmissive reflective films [3], flat panel display reflective electrode films and conductive films [5,6,7,8,9]. Silver also has good conductivity, high thermal conductivity, high reflectivity, anti-welding, a low extinction coefficient, good surface smoothing, good adhesion and other excellent features; hence it is widely used in electrical contact materials [10], welding materials [11], silver paste [12], the energy industry [13,14,15] and so on.

However, problems regarding pure silver, such as being easily affected by sulfide corrosion and oxidization, have seriously affected the performance and use of silver products [16,17,18]. Therefore, researchers often use alloying elements or surface modification to improve silver processing and weather ability [19,20]. Zhao et al. [21] enhanced the anti-perspiration liquid silver-based alloy corrosion resistance and anti-sulfur silver alloy discoloration ability by substituting Cu of sterling silver with 0.375 wt.% In. Cao et al. [22] studied the effect of Au on the homogenization of the Al–Cu alloy. The addition of trace Au affected the distribution of Cu atoms in the Al–Cu alloy but caused the segregation of Cu. The effect of Ge on the microstructure and properties of 925 silver has also been studied. Bo et al. [23] reported that when the content of Ge was 0.5%, the as-cast hardness of the alloy was higher and the cold working performance, electrochemical corrosion resistance and color change resistance were better. However, up until now, no publications have reported on the microstructures and property changes of Ag-1.5Cu-0.1Y alloys with the addition of Au and Ge.

Therefore, in this study, we attempted to explore the effects of Au and Ge on the microstructures and properties of Ag-1.5Cu-0.1Y alloys. The corrosion resistance, microhardness and electrical resistivity of these alloys were tested. It was demonstrated that the microstructures were refined in Ag-1.5Cu-0.1Y alloys with Au and Ge added. The corrosion resistance, microhardness and electrical resistivity of Ag-1.5Cu-0.1Y alloys with the addition of Au and Ge were enhanced. This work will provide an important basis for the subsequent preparation of high quality and high-performance silver-based alloys.

## 2. Materials and Methods

### 2.1. Alloy Design and Preparation

The alloys of Ag-1.5Cu-0.1Y-*x*Au-*y*Ge (*x* = 0, 0.5, 1.0, 1.5; *y* = 0, 0.5, 1.0, 1.5) used in this study were prepared from high-purity 99.99 wt.% Ag, Cu, Y, Au and Ge based on the alloy Ag-1.5Cu-0.1Y (Table 1). These alloys were prepared by a self-designed open high-frequency induction furnace equipped with an argon (99.99%) atmosphere protection device at 0.1 MPa. First, the pure Cu and Y were wrapped in the melting pure Ag foils, which then were added to the graphite ceramic crucible at 1873 K for 10 min. After all the elements had melted, the graphite crucible was delivered to the casting area, then the melted alloy was poured into a cast-iron mold to produce tensile bars of Φ 9 mm × 90 mm. The alloy was stirred at 900 rpm/min for three minutes by a permanent magnetic stirring device, followed by water quenching until the alloy solidified. The alloy was protected in the top thermal and Argon atmosphere at all times during the cooling process. The cooled tensile bars of all alloys were used in the following analysis.

### 2.2. Microstructures and Properties Testing

For the microstructure observations, samples were wet ground with silicon carbide abrasive papers from P800 to P5000, followed by polishing with a light MgO suspension solution. A fresh corrosive liquid (30% H_2_O_2_: 25% NH_3_·H_2_O = 1:3) was used to analyze the constituents of the alloys. The microstructures observations were conducted on an optical microscope Leica DMRX (Leica, Wetzlar, Germany), and phase analysis was conducted by a D8 ADVANCE X-ray diffractometer (XRD) (Bruker, Madison, WI, USA). A JXA-8530F electron probe microanalyzer (EPMA) (JEOL, Tokyo, Japan) was used to further assess the microstructures and the elemental composition analysis of the alloy samples. Microhardness tests were performed on an HXS-1000AK microhardness tester (Jingda, Xi’an, China) with a load of 0.245 N and dwelling time of 10 s at room temperature. Electrical resistivity tests were carried out on a ZY9858 digital micro-ohmmeter (Zhengyang, Shanghai, China) at room temperature. Corrosion resistance tests were conducted in a closed container with H_2_S and the changes were observed at 0.5, 1, 2, 4, 6 and 8 h.

## 3. Results and Discussion

### 3.1. Microstructures

**Figure 1:**
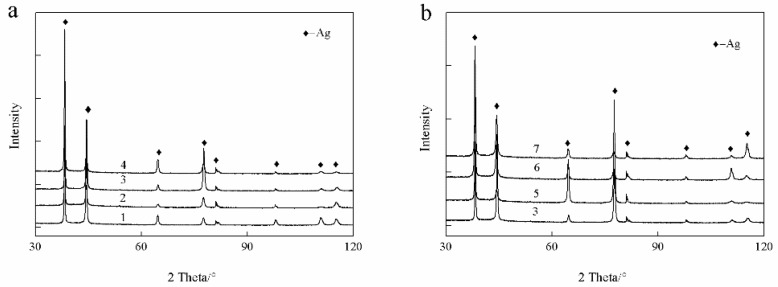
X-ray diffractometer (XRD) patterns of the Ag-1.5Cu-0.1Y alloys with different additions of the elements Au (**a**) and Ge (**b**).

In this study, a small amount of Au and Ge was added to the Ag-1.5Cu-0.1Y alloys in an attempt to improve the properties. Generally, the properties of metal materials may vary greatly with the different compositions and microstructures. Therefore, we first analyzed the XRD phase of the Ag-1.5Cu-0.1Y alloys with different quantities of Au and Ge added. The results showed that only the α-phase silver existed in the XRD patterns of the Ag-1.5Cu-0.1Y alloy with the addition of Au and Ge (Figure 1). No new diffraction peak appeared in the Ag-1.5Cu-0.1Y alloy with the increased content of Au and Ge, which indicated that Au and Ge may be in the form of solute atoms in the solid solution (primary solid solution) or in the form of secondary phases (secondary solid solutions). This abovementioned evidence indicates that the addition of Au and Ge had little effect on the phase of the Ag-1.5Cu-0.1Y alloy, which may be due to the little amounts of Au and Ge added in the study (Au: 0–1.5 wt.%; Ge: 0–1.5 wt.%).

**Figure 2:**
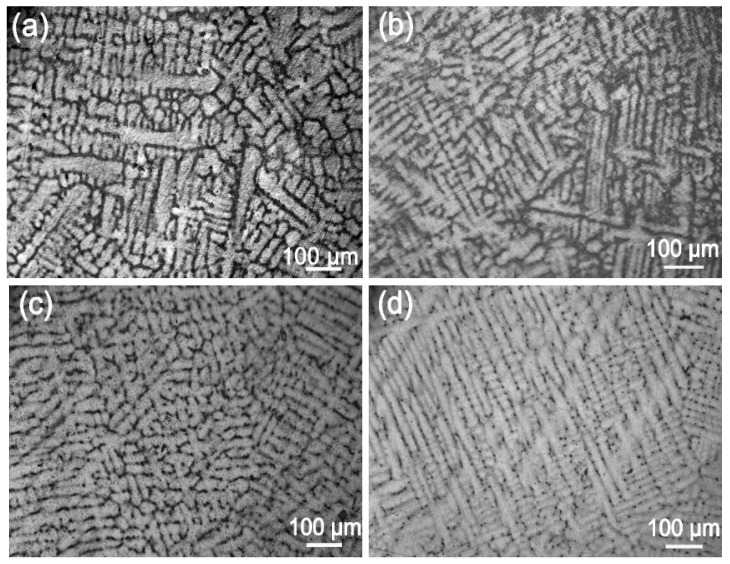
Microstructures of the Ag-1.5Cu-0.1Y alloys with different quantities of the element Au: (**a**) 0 wt.%; (**b**) 0.5 wt.%; (**c**) 1.0 wt.%; and (**d**) 1.5 wt.%.

**Figure 3:**
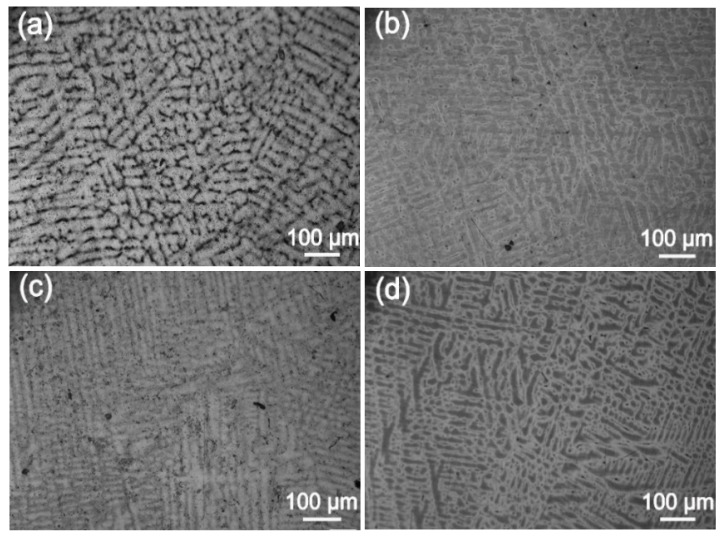
Microstructures of the Ag-1.5Cu-0.1Y-1.0Au alloys with different quantities of the element Ge: (**a**) 0 wt.%; (**b**) 0.5 wt.%; (**c**) 1.0 wt.%; and (**d**) 1.5 wt.%.

**Figure 4:**
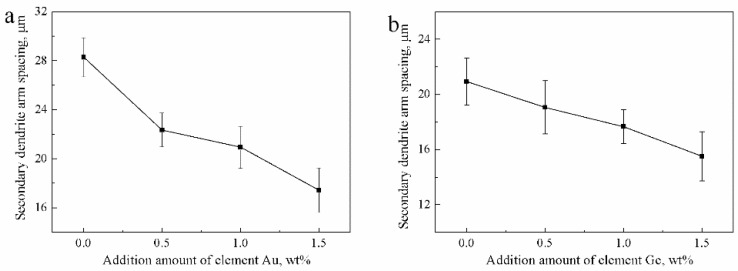
Secondary dendrite arm spacing of the Ag-1.5Cu-0.1Y alloys with different additions of Au and Ge: (**a**) Ag-1.5Cu-0.1Y-*x*Au alloys and (**b**) Ag-1.5Cu-0.1Y-1.0Au-*x*Ge alloys (*x =* 0–1.5). The error bars represent the standard deviation of the five replicates.

Next, we analyzed the microstructures of the Ag-1.5Cu-0.1Y alloys with different quantities of Au and Ge added. The crystal structure showed a typical dendrite structure, and the grains were composed of columnar crystals and equiaxed grains formed after dendrite fusing (Figure 2 and Figure 3). The dendrites of the Ag-1.5Cu-0.1Y alloys with Au and Ge added were finer than those with no addition of Au and Ge. Furthermore, with the increase in the addition of Au and Ge, the secondary dendrite arm became shorter and the secondary dendrite arm spacing (λ_2_) became smaller (Figure 4), which indicated that the structure of the Ag-1.5Cu-0.1Y alloys with Au and Ge added were more compact. In addition, the secondary dendrite arm spacing of the Ag-1.5Cu-0.1Y alloys with Ge was smaller and denser than that of Ag-1.5Cu-0.1Y alloys with Au (Figure 4), which demonstrated that the microstructures were more refined in the Ag-1.5Cu-0.1Y alloys with two elements (Au and Ge) added when compared with the alloy samples with only Au added. The most obvious difference between the two types of silver-based alloys was that the grain boundary of the Ag-1.5Cu-0.1Y-1.0Au alloys with Ge added became brighter and the crystal darkened after the metallographic etching, whereas the Ag-1.5Cu-0.1Y alloys with only Au added was demonstrated to always be brighter. This may be because the addition of Ge can form an oxide film on the surface of the Ag-1.5Cu-0.1Y alloys, which may increase the oxidation resistance and vulcanization resistance at the grain boundary. However, with the increase of Au and Ge (Au: 1.5 wt.%, Ge: 1.5 wt.%), black aggregates appeared in the grain boundary of the Ag-1.5Cu-0.1Y alloys (Figure 2d and Figure 3d).

**Figure 5:**
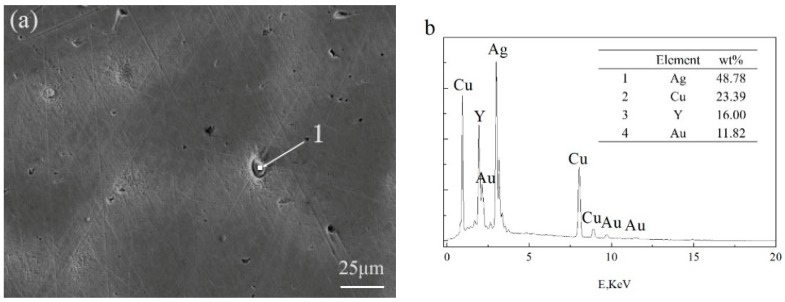
Scanning electron microscope (SEM) (**a**) and EDS (**b**) analysis of the black agglomerate in the Ag-1.5Cu-0.1Y-1.5Au alloy.

**Figure 6:**
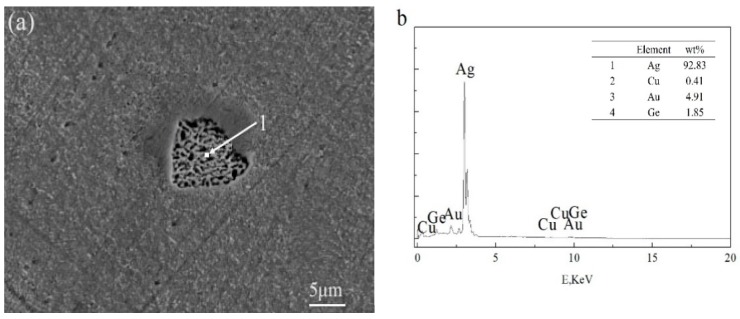
SEM (**a**) and EDS (**b**) analysis of black agglomerate in the Ag-1.5Cu-0.1Y-1.5Au-1.5Ge alloy.

The appearance of black aggregates may affect the properties of a Ag-1.5Cu-0.1Y alloy. Therefore, we further analyzed the composition of the black aggregates. The results of the energy dispersive spectrometer (EDS) analysis of the dendrites and intergranular of Ag-1.5Cu-0.1Y alloys with different Au and Ge additions are shown in Table 2 and Table 3, and the black agglomerations are shown in Figure 5 and Figure 6, respectively. When the addition of Au reached 0.5 wt.%, the presence of Au was detected at the grain boundary, but the Au was not detected in the crystal (Table 2). This was also the case in the Ag-1.5Cu-0.1Y-1.0Au alloys with 0.5 wt.% Ge added (Table 3). This may be due to the low addition of Au and Ge resulting in a small amount of the Au and Ge solid solution in the alloy crystal, which was not easy to detect. Au was detected in both the intragranular and grain boundaries of the Ag-1.5Cu-0.1Y alloys with an Au content of 1.0 wt.% and 1.5 wt.%, and there was little difference between intragranular and intergranular contents (Table 2). These data also indicated that the Au was soluble in the alloy structure, which was consistent with the content of the Ag–Cu alloy being able to form a continuous solid solution phase and infinite solid solution [24]. The EDS results showed that the black aggregates were mainly composed of 48.78 wt.% Ag, 23.39 wt.% Cu, 16.00 wt.% Y and 11.82 wt.% Au (Figure 5b). Since the XRD analysis results of the Ag-1.5Cu-0.1Y-1.5Au alloy (Figure 1a) found no new diffraction peak, this means that the black aggregates were the aggregates of Cu, Y and Au elements and not a new intermetallic compound. Ge elements were also detected in both the intragranular and grain boundaries in the Ag-1.5Cu-0.1Y-1.0Au alloys with 1.0 wt.% and 1.5 wt.% Ge added (Table 3). The EDS results showed that the black aggregates of Ag-1.5Cu-0.1Y-1.0Au-1.5Ge were Cu, Y, Au and Ge aggregates composed of 92.83 wt.% Ag, 0.41 wt.% Cu, 4.91 wt.% Au and 1.85 wt.% Ge (Figure 6b). It was reported that the maximum solid solubility of Ge in Ag at the eutectic point (651 °C) was 9.6% (6.68 wt.%) [25], the appearance of black aggregates might be because 1.5% wt.% Ge was not dissolved well enough in Ag.

### 3.2. Corrosion Resistance

**Figure 7:**
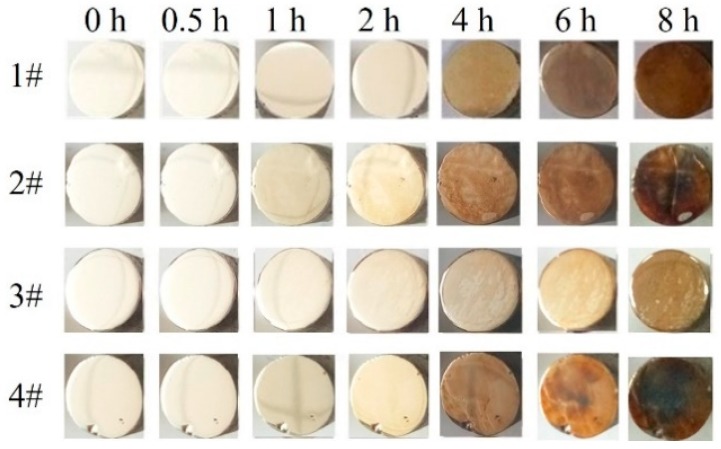
Corrosion resistance of Ag-1.5Cu-0.1Y alloys with different additions of Au.

The corrosion resistance of the Ag-1.5Cu-0.1Y alloys with Au added increased when compared with the Ag-1.5Cu-0.1Y alloy with no Au added (Figure 7). The Ag-1.5Cu-0.1Y alloy with an Au amount of 1.0 wt.% had the best corrosion resistance among the four samples, and its surface began to become slightly yellow when the corrosion resistance experiment was carried out to 4 h. The Ag-1.5Cu-0.1Y alloys with 0.5 wt.% and 1.5 wt.% Au added began to become yellowish when the vulcanization test was carried out for 2 h, and the corrosion resistance was worse than that with 1.0 wt.% Au. The corrosion resistance increases of the Ag-1.5Cu-0.1Y alloys with Au added could be explained by the fact that the electrode potential was higher in Au (1.692 V) than in Ag (0.799 V). The addition of Au increased the electrode potential of the Ag-1.5Cu-0.1Y alloy, and the vulcanization reaction was mainly an electrochemical corrosion. Therefore, adding Au can significantly improve the corrosion resistance of the Ag-1.5Cu-0.1Y alloy. The lower corrosion resistance of Ag-1.5Cu-0.1Y-1.5Au than Ag-1.5Cu-0.1Y-1.0Au could be explained by the fact that aggregates containing a lot of Cu and Y elements appeared in the matrix of the Ag-1.5Cu-0.1Y-1.5Au alloy, so the lower electrode potential of elements Cu and Y resulted in selective vulcanization [26].

**Figure 8:**
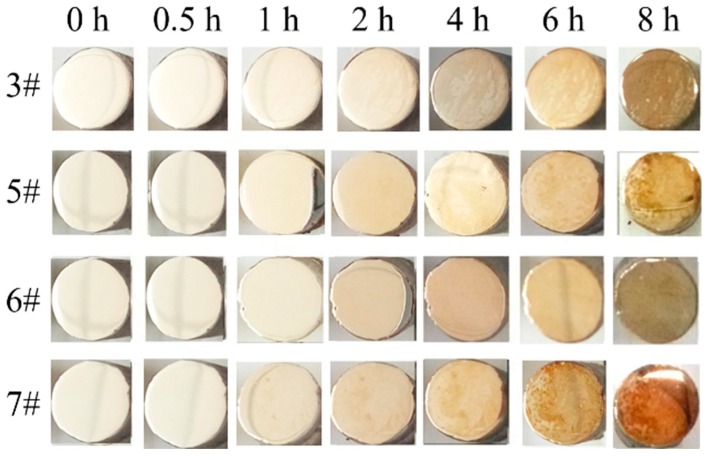
Corrosion resistance of the Ag-1.5Cu-0.1Y-1.0Au alloys with different additions of Ge.

Considering the fact that silver-based alloys are mainly used in reflective films, it is necessary to keep the vulcanization resistance of the silver film. Therefore, to investigate the corrosion resistance of the Ag-1.5Cu-0.1Y alloy with Ge added, the addition of Au was optimized by 1.0 wt.%. The corrosion resistance of the Ag-1.5Cu-0.1Y alloy with Ge added was better than that of Sample 3 (Ag-1.5Cu-0.1Y-1.0Au) without adding Ge (Figure 8). Moreover, Sample 6 with the Ge addition of 1.0 wt.% had the best corrosion resistance out of all the samples with Ge added (Figure 8). The corrosion resistance mechanism of Ge can be explained by the Ge electrode potential (0.12 V) being lower than that of Ag (0.799 V). Ge can spontaneously form a passivation film on the surface of the Ag-1.5Cu-0.1Y alloy to slow down the corrosion of the inner layer and to protect the body. Meanwhile, Ge and S will prioritize the formation of grey GeS_2_, thus postponing the silver vulcanization reaction to produce Ag_2_S and increasing the anti-sulfur discoloring property of the Ag-1.5Cu-0.1Y alloy as well as inhibiting the decrease in reflectivity.

### 3.3. Microhardness

The mechanical properties, wear resistance and yield strength of the material have a direct relationship with the microhardness of the material [27]. Therefore, the measurement of the microhardness of the sample can be effective in evaluating the properties of the material. 

**Figure 9:**
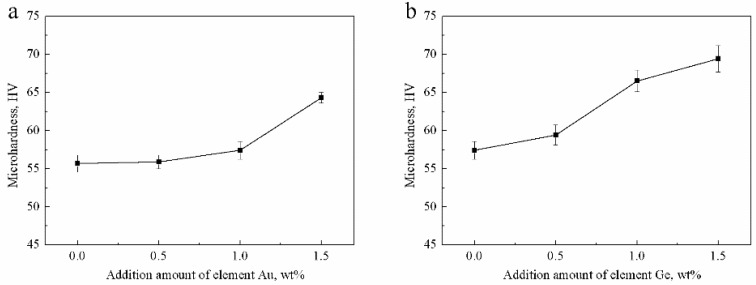
Microhardness of the Ag-1.5Cu-0.1Y alloys with different Au and Ge additions in (**a**) Ag-1.5Cu-0.1Y-*x*Au alloys and (**b**) Ag-1.5Cu-0.1Y-1.0Au-*x*Ge alloys (*x =* 0–1.5). The error bars represent the standard deviation of the six replicates.

The microhardness of four Ag-1.5Cu-0.1Y-*x*Au alloy samples (*x* = 0, 0.5, 1.0, 1.5) were 55.68 ± 1.14, 55.89 ± 0.91, 57.42 ± 1.16 and 64.34 ± 0.70 HV, respectively (Figure 9a). With the increase in the addition of Au, the microhardness of the Ag-1.5Cu-0.1Y alloy increased. The increased microhardness may be explained by the solid solution strengthening of the Ag-1.5Cu-0.1Y alloy by Au; as more Au was added, the greater the strengthening effect of the solid solution. However, Au was not detected in the crystal of Ag-1.5Cu-0.1Y-0.5Au, which indicated that the distribution of Au existed in the form of segregation. Au content in the solid solution decreased and the strengthening of the solid solution played a major role in the hardness degree when there were less alloy elements, which may have led to a decrease in the Ag-1.5Cu-0.1Y-0.5Au alloy microhardness. In summary, the greater the amount of Au added, the higher the microhardness of the Ag-1.5Cu-0.1Y alloys. 

The microhardness of the four Ag-1.5Cu-0.1Y-1.0Au-*x*Ge alloy samples (*x* = 0, 0.5, 1.0 and 1.5) were 57.42 ± 1.16, 59.43 ±1.31, 66.53 ± 1.40 and 69.45 ± 1.73 HV, respectively (Figure 9b). Similarly, with the increase of Ge content, the microhardness Ag-1.5Cu-0.1Y-1.0Au-*x*Ge was continuously improved. The main strengthening methods of Ge were also solid solution strengthening. The solid solution strengthening parameters of Cu and Ge for silver were 0.195 and 0.225, respectively, and the difference in relative atoms in Cu and Ag and in Ge and Ag were 11.50% and 15.30%, respectively [28]. Therefore, the easily formed solid solution made the crystal grain more refined, the microstructure more compact and the lattice distortion increased. Then, the formation of a high-density dislocation resulted in the increased microhardness of the Ag-1.5Cu-0.1Y alloy. In addition, the black agglomerates on the grain boundaries, resulting from the excess Ge added, also led to the increased microhardness of the Ag-1.5Cu-0.1Y alloy. The relationship between the microhardness and λ_2_ can be expressed by the following Hall–Petch equation [29].

(1) $$\mathrm{HV}={\mathrm{HV}}_{0}+k\lambda_{2}^{-0.5}$$

where HV_0_ and *k* are material constants.

**Figure 10:**
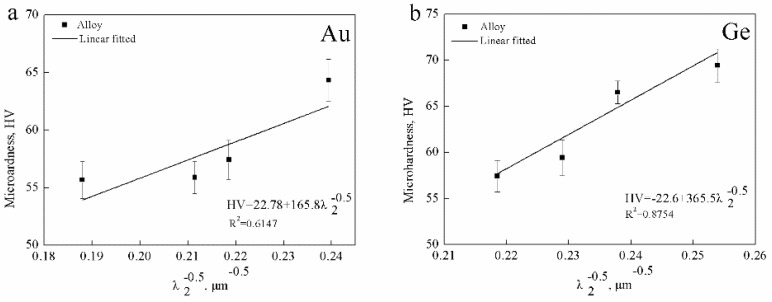
Linear fitted between the microhardness and the λ_2_ of (**a**) Ag-1.5Cu-0.1Y-*x*Au and (**b**) Ag-1.5Cu-0.1Y-1.0Au-*x*Ge (*x* = 0–1.5).

Figure 10 shows the relationship between the hardness and λ_2_ of the Ag-1.5Cu-0.1Y alloys with different Au and Ge added. It can be seen that there was a certain correlation between the microhardness and λ_2_, where the smaller the λ_2_, the higher the microhardness. Additionally, a higher relevance between the microhardness and λ_2_ was found in the Ag-1.5Cu-0.1Y-1.0Au alloys with Ge added than in the alloy samples (Ag-1.5Cu-0.1Y) with Au added; however, the correlation between the microhardness and λ_2_ in Ag-1.5Cu-0.1Y with Au added was relatively low (R_Au_^2^ = 0.6147, R_Ge_^2^ = 0.8754) (Figure 10).

### 3.4. Electrical Conductivity

Electrical resistivity is an electrical parameter indicating the resistance of a conductor to the current through its characteristics. It is independent of the length of the alloy material and the cross-section area. It is related to its chemical composition, metallographic structure and working temperature [30]. Silver-based films need good electrical conductivity, so the conductivity of the alloy samples needed to be tested to ensure the good conductivity of the silver-based films prepared by magnetron sputtering.

**Figure 11:**
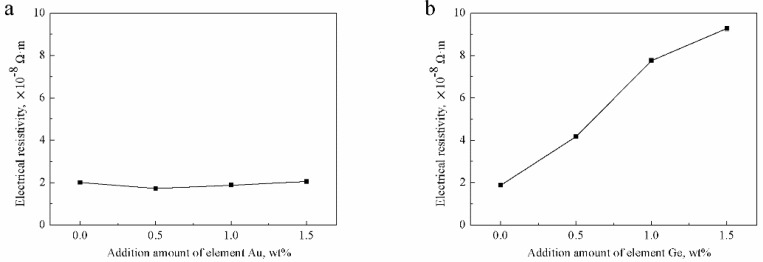
Electrical resistivity of the Ag-1.5Cu-0.1Y alloys with different additions of Au and Ge in (**a**) Ag-1.5Cu-0.1Y-*x*Au alloys and (**b**) Ag-1.5Cu-0.1Y-1.0Au-*x*Ge alloys (*x =* 0–1.5). The error bars represent the standard deviation of the three replicates.

The electrical resistivity of the Ag-1.5Cu-0.1Y alloys with the addition of 0.5 wt.% and 1.0 wt.% Au were lower than that of the no Au sample, whereas the electrical resistivity of the Ag-1.5Cu-0.1Y alloy with 1.5 wt.% added was slightly higher than that of the without Au added sample (Figure 11a). With the increase in the addition of Ge, the electrical resistivity of the Ag-1.5Cu-0.1Y alloy increased, leading to the decrease in the conductivity of the Ag-1.5Cu-0.1Y alloys (Figure 11b). The above data indicated that adding a small amount of Au could improve the conductivity of the Ag-1.5Cu-0.1Y alloy; however, adding too much Au reduced the conductivity of the Ag-1.5Cu-0.1Y alloy.

The electrical resistivity of the Ag-1.5Cu-0.1Y alloy with the addition of 0.5 wt.% Au was slightly decreased, which can be explained by two aspects: First, the low electrical resistivity of pure Au (2.40 × 10^−8^ Ω∙m) resulted in a less affected electrical resistivity of the alloy; and second, the small amount of Au added fixed other elements, improved the uniformity of the structure and composition and then reduced the number of precipitated phases, thus reducing the electrical resistivity However, the rich Cu and Au agglomerate that appeared had an effect on the electrical resistivity of the Ag-1.5Cu-0.1Y-1.5Au alloys and reduced the conductivity. The increased electrical resistivity of Ag-1.5Cu-0.1Y-1.0Au-*x*Ge could be explained as follows: Ge is a semiconductor element, so its own electrical resistivity is high (0.47 Ω∙m); therefore, when the black aggregates (Ag0.41Cu4.91Au1.85Ge) increased at the grain boundary when the addition of Ge increased, this produced the electronic wave scattering in these defects and increased the electrical resistivity and reduced the electrical conductivity of the alloy. At the same time, with the increase in the addition of Ge, the dendrites were more refined, the number of grain boundaries increased and the free path of electrons was limited, which affected the electrical conductivity of the alloys [31,32]. Taken together, the addition of Ge had a great influence on the conductivity of the Ag-1.5Cu-0.1Y alloys; therefore, it could maintain the conductive properties of the Ag-1.5Cu-0.1Y alloy by controlling the amount of Ge added.

## 4. Conclusions

The addition of Au and Ge refined the crystal grains and shortened the secondary dendrites of the Ag-1.5Cu-0.1Y alloy, mainly in the form of a solid solution in the Ag-1.5Cu-0.1Y alloy. The addition of 1.5 wt.% Au and Ge may not dissolve well in the Ag-1.5Cu-0.1Y alloy, thus, few black aggregates would appear at the grain boundary. The refined microstructures altered the properties of the Ag-1.5Cu-0.1Y alloys. The corrosion resistance of the Ag-1.5Cu-0.1Y alloys was enhanced by the addition of Au and Ge, and the addition of 1.0 wt.% Au and Ge showed the best corrosion resistance in the Ag-1.5Cu-0.1Y alloys. The microhardness of the Ag-1.5Cu-0.1Y alloys increased with the increase in Au or Ge content. The electrical resistivity of the Ag-1.5Cu-0.1Y alloys increased with the addition of Ge. Nevertheless, it could not be significantly changed by Au.

## Figures and Tables

**Table 1:** Chemical composition of the Ag-1.5Cu-0.1Y-*x*Au-*y*Ge alloys (wt.%).

Sample ID	Ag	Cu	Y	Au	Ge
1#	98.4	1.5	0.1	0	0
2#	97.9	1.5	0.1	0.5	0
3#	97.4	1.5	0.1	1.0	0
4#	96.9	1.5	0.1	1.5	0
5#	96.9	1.5	0.1	1.0	0.5
6#	96.4	1.5	0.1	1.0	1.0
7#	95.9	1.5	0.1	1.0	1.5

**Table 2:** Energy dispersive spectrometer (EDS) analysis results of the Ag-1.5Cu-0.1Y-*x*Au (*x* = 0, 0.5, 1.0, 1.5) alloys.

Sample ID	Ag		Cu		Y		Au	
	a	b	a	b	a	b	a	b
1#	99.12	94.06	0.88	3.35	0	2.59	-	-
2#	98.91	91.86	1.09	4.97	0	1.42	0	1.74
3#	91.20	95.33	0.71	2.70	0	0.94	1.10	1.03
4#	97.61	92.05	0.82	5.52	0	0.79	1.57	1.64

Note: **a** represents intergranular and **b** represents intragranular.

**Table 3:** EDS analysis of the Ag-1.5Cu-0.1Y-1.0Au-*x*Ge (*x* = 0, 0.5, 1.0, 1.5) alloys.

Sample ID	Ag		Cu		Y		Au		Ge	
	a	b	a	b	a	b	a	b	a	b
**3#**	91.20	95.33	0.71	2.70	0	0.94	1.10	1.03	-	-
**5#**	97.08	92.67	1.43	3.04	0	0	1.49	1.67	0	2.62
**6#**	96.67	95.31	1.09	1.81	0	0	1.85	1.98	0.39	0.90
**7#**	97.51	96.76	0.74	1.16	0	0	1.27	1.03	0.48	1.04

Note: **a** represents intergranular and **b** represents intragranular.
